# Cranio-Caudal Kinematic Turn Signature Assessed with Inertial Systems As a Marker of Mobility Deficits in Parkinson’s Disease

**DOI:** 10.3389/fneur.2018.00022

**Published:** 2018-01-29

**Authors:** Karina Lebel, Christian Duval, Hung Phuc Nguyen, Réjean Plamondon, Patrick Boissy

**Affiliations:** ^1^Department of Surgery, Faculty of Medicine and Health Sciences, Orthopedic Service, Université de Sherbrooke, Sherbrooke, QC, Canada; ^2^Research Centre on Aging, Sherbrooke, QC, Canada; ^3^Département des Sciences de l’activité Physique, Université du Québec à Montréal, Montreal, QC, Canada; ^4^Centre de Recherche Institut Universitaire de Gériatrie de Montréal, Montreal, QC, Canada; ^5^Laboratoire Scribens, Département de génie Électrique, École Polytechnique de Montréal, Montréal, QC, Canada

**Keywords:** turn, deficit, signature, inertial motion capture, inertial measurement unit, AHRS, Parkinson’s disease, sigma-lognormal

## Abstract

**Background:**

Turning is a challenging mobility task requiring proper planning, coordination, and postural stability to be executed efficiently. Turn deficits can impair mobility and lead to falls in patients with neurodegenerative disease, such as Parkinson’s disease (PD). It was previously shown that the cranio-caudal sequence involved during a turn (i.e., motion is initiated by the head, followed by the trunk) exhibits a signature that can be captured using an inertial system and analyzed through the Kinematics Theory. The so-called cranio-caudal kinematic turn signature (CCKS) metrics derived from this approach could, therefore, be a promising avenue to develop and track markers to measure early mobility deficits.

**Objective:**

The current study aims at exploring the discriminative validity and sensitivity of CCKS metrics extracted during turning tasks performed by patients with PD.

**Methods:**

Thirty-one participants (16 asymptomatic older adults (OA): mean age = 69.1 ± 7.5 years old; 15 OA diagnosed with early PD ON and OFF medication, mean age = 65.8 ± 8.4 years old) performed repeated timed up-and–go (TUG) tasks while wearing a portable inertial system. CCKS metrics (maximum head to trunk angle reached and commanded amplitudes of the head to trunk neuromuscular system, estimated from a sigma-lognormal model) were extracted from kinematic data recorded during the turn phase of the TUG tasks. For comparison purposes, common metrics used to analyze the quality of a turn using inertial systems were also calculated over the same trials (i.e., the number of steps required to complete the turn and the turn mean and maximum velocities).

**Results:**

All CCKS metrics discriminated between OA and patients (*p* ≤ 0.041) and were sensitive to change in PD medication state (*p* ≤ 0.033). Common metrics were also able to discriminate between OA and patients (*p* < 0.014), but they were unable to capture the change in medication state this early in the disease (*p* ≥ 0.173).

**Conclusion:**

The enhanced sensitivity to change of the proposed CCKS metrics suggests a potential use of these metrics for mobility impairments identification and fluctuation assessment, even in the early stages of the disease.

## Introduction

Idiopathic Parkinson’s disease (PD) is a progressive neurodegenerative disease marked by the loss of neurons producing dopamine. Symptoms of PD include tremor, muscle rigidity, postural instability, akinesia (lack of movement), and bradykinesia (slowness of movement) (1). The ability of individuals with PD to move around their environment and execute functional activities of daily living is, therefore, jeopardized by the symptoms associated with the disease. As a result, impaired mobility is recognized as a major cause of disability for patients, affecting their quality of life (2, 3). Mobility deficits also contribute to the occurrence of adverse events such as falls. Falls are common events among patients with PD, with approximately two-thirds of them being fallers (4, 5). It has been shown that most falls are due to patient-related factors (i.e., intrinsic factors) and occur mainly while turning (6). To alleviate PD symptoms and related deficits, medication remains the principal intervention (7). However, to be efficient, medication type and dosage have to be personalized according to the evolution of symptoms. Such personalized process requires precise information on the patient’s ability to function during daily living. Unfortunately, current clinical scales provide only a limited portrait of the impact of symptoms, often neglecting the variations with time and associated movement while exhibiting a limited sensitivity to change (8, 9). Our research, therefore, aims at developing objective indicator, also called biomarkers, enabling the assessment of the patients’ ability to perform mobility and functional activities as the disease progresses.

Mobility deficits have been traditionally studied and evaluated in motion laboratory, using advanced equipment such as camera-based optoelectronic devices (10–13). Using such setup, it was shown that specific characteristics of a turn are affected in individuals with PD, even at early stages of the disease (14). Patients tend to turn slower, with an increased number of steps and to use different motor strategies. Among these strategies, the so-called cranio-caudal strategy was shown to be altered in patients with PD. The cranio-caudal strategy refers to a specific cranio-caudal sequence of motions typically exhibited during a turn in healthy individuals (12, 15): the motion is initiated by the head, followed by the trunk and the pelvis until the body’s reorientation process toward the new direction to pursue is completed. This sequence can be seen as a specific cranio-caudal turn signature, which concept can be defined as the specific way (timing, force, amplitude, velocity) a movement is performed. Using laboratory equipment, it was shown that patients with PD exhibit an increased coupling of the rotational axis during the turn, as assessed with a decreased maximum angle of the head relative to the trunk (11–13), and that they initiate the turn later than healthy controls (10). Although, these variables offer unique insights into motor coordination of PD patients during turning, the usability and clinical applicability of the motion-capture measurement approach used to obtain these variables limit their use as outcomes measures (16).

Over the past decade, wearable inertial systems (IS) have emerged as an alternative to traditional motion-capture system for clinical applications. IS are composed of inertial measurement units (IMU) which include accelerometers, gyroscopes, and magnetometers to measure linear acceleration, angular velocity, and magnetic field, respectively. Nowadays, most IS also include a fusion algorithm which estimates the 3D orientation of the IMU, enabling the capture of motion. Recently, we proposed a measurement approach using measures of trunk and head motions captured with IS to identify cranio-caudal kinematic turn signature (CCKS) metrics (17). On healthy individuals, the approach showed a good ability to capture the turn signature and the metrics derived were proven to be robust to speed variations and reliable (17). The objectives of the present study were (i) to explore the ability of the CCKS turn metrics measured using wearable inertial systems (IS) to discriminate between healthy and individuals with PD, (ii) to investigate the sensitivity to change of CCKS turn metrics during ON and OFF medication states in these patients who have been recently diagnosed with PD, and (iii) to assess the results in comparison to traditional markers of turn. This manuscript first introduces the CCKS technique and then describes the sample and protocol used to meet the specific objective of the paper. Results regarding both the discriminative validity and the sensitivity to change of the CCKS metrics and the traditional turn metrics are then presented, followed by a discussion on the findings.

## Materials and Methods

### Turn Cranio-Caudal Kinematic Signature from IS

Assessment of the turn cranio-caudal kinematic signature refers to the characterization of the axial motion of the head relative to the trunk during the turn. Healthy individuals initiate a turn with the head, followed by the trunk and then the pelvis. The turn cranio-caudal movement can, therefore, be divided into two main phases: (i) the head moves away from the trunk, initiating the turn task; and (ii) the trunk engages into the turn closely followed by the pelvis, closing the gap with the head as the body realigns toward the new desired direction. Assessing the signature of that motion enables identification of different strategies and their related deficits.

**Figure 1:**
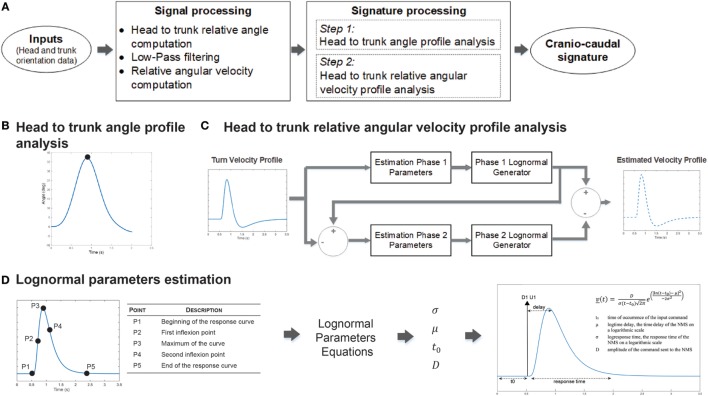
Cranio-caudal kinematics signature processing. **(A)** Post-processing analysis overview. Head to trunk relative orientation during the turn was computed and derived to obtain the associated head to trunk relative angular velocity profile. Both signals are then used for signature recognition. **(B)** Head to trunk angular profile is analyzed to determine the maximum relative angle reached during the turn. **(C)** Head to trunk relative angular velocity profile analysis considers the turn as a two-phase movement. At turn initiation, a first command is sent to the neuromuscular system (NMS) to initiate the motion of the head. Shortly after, trunk motion is initiated through a second command. The difference in the NMS responses to those two commands generates the observed head to trunk velocity profile. Analysis of this profile, therefore, derives the lognormal parameters associated with the first phase, uses the lognormal equations on these parameters to deduce the remaining profile and, hence, the associated phase 2 lognormal parameters. **(D)** Lognormal parameters are derived from specific points located on the velocity profile curve, which coordinates are used in combination to lognormal parameters equations.

The proposed cranio-caudal signature analysis is a two-step process, as presented in Figure 1A, and detailed in Lebel et al. (17). First, the head to trunk angular profile is evaluated to determine the maximum angle reached during the turn (Figure 1B). Then, a model based on the kinematic theory, the sigma-lognormal model, is used to get insights into the participant’s neuromuscular system (NMS) (Figure 1C). Briefly, the kinematic theory stipulates that the response of the NMS can be recognized from the characteristics of the movement itself (18) (Figure 1D). Hence, the head to trunk relative angular velocity profile is analyzed with the sigma-lognormal mathematical approach, enabling the deduction of some of the participant’s NMS response characteristics. Specifically, for each phase of the turn, the model allows to determine.

- D: the amplitude of the command sent to the NMS,
- t_0_: the time of occurrence of the command,
- μ: the time delay of the NMS on a logarithmic scale,
- σ: the response time of the NMS on a logarithmic scale.

Mathematical details for the derivation of the different sigma-lognormal parameters as well as further details on the cranio-caudal turn kinematic signature characterization method are available in Lebel et al. (17).

**Figure 2:**
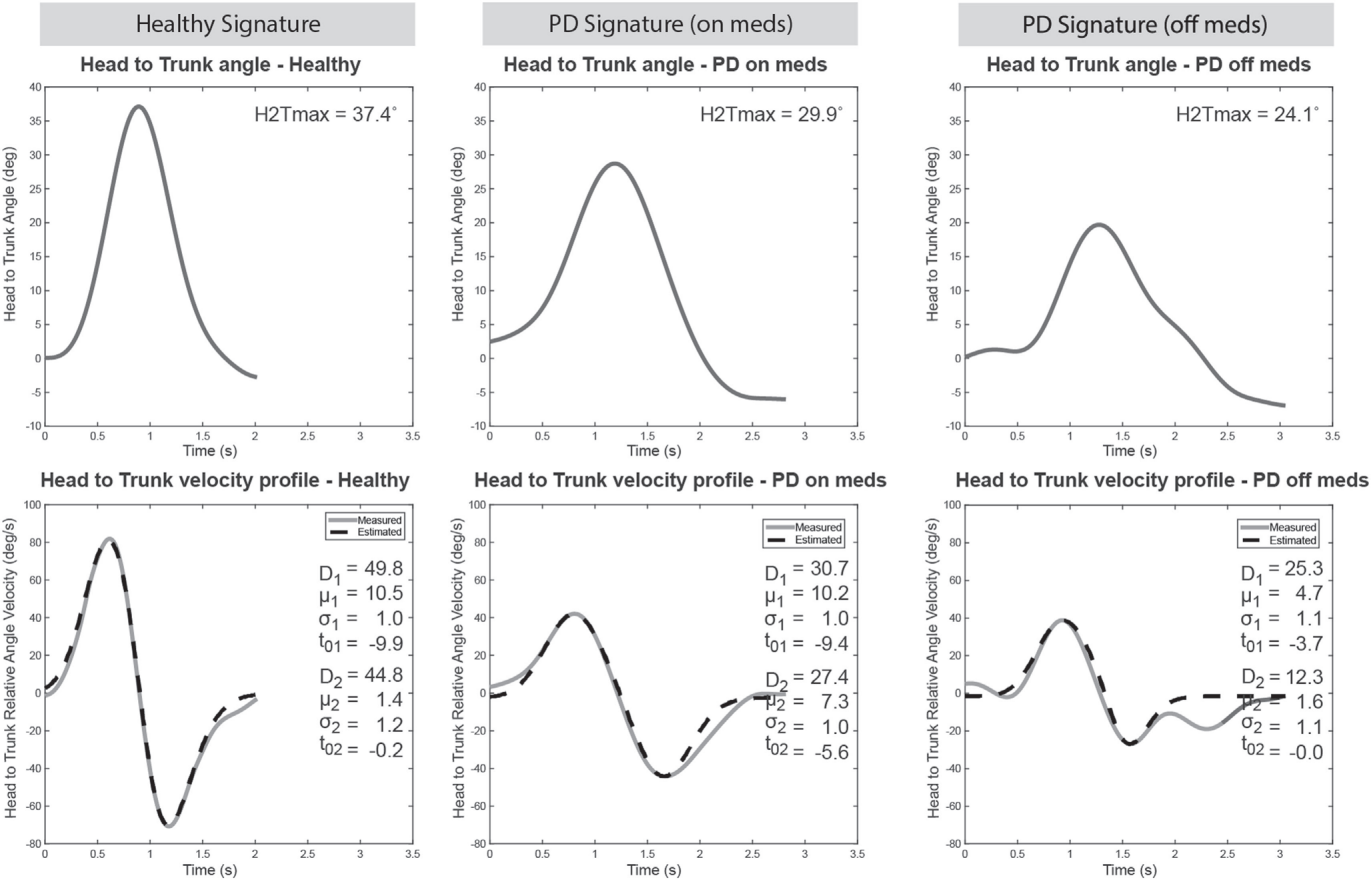
Cranio-caudal kinematic turn signature typical results. Example of signature patterns for a healthy elderly (left panels), a Parkinson’s disease (PD) patient on medication (middle panels) and that same PD patient off medication (right panels). The top panels show the variation in relative orientation of the head to the trunk during the turn. The maximum head to trunk angle (H2Tmax) is derived from it as a metric. The bottom panels illustrate the relative angular velocity profile (solid line) along with the estimated profile obtained with the sigma-lognormal model analysis (dotted line). Parameters of the sigma-lognormal are shown as inserts.

Figure 2 shows typical graph results for the cranio-caudal signature found in a healthy elderly individual, patient with PD *ON* medication, and the same patient *OFF* medication. The present study concentrates on differences in signature patterns to appraise the ability of the approach to discriminate between patients and healthy controls and to determine its sensitivity to change. Specifically, this study focusses on the variation of the maximum head to trunk angle and the amplitude of the NMS commands for both turn phases as these parameters have shown the highest reliability (17).

### Participants

Thirty-one participants [16 asymptomatic older adults (OA) aged between 55 and 83 years old and 15 OA diagnosed with Parkinson’s disease (PD), aged between 56 and 79 years old] were recruited from the community in collaboration with Quebec Parkinson Network. A description of the sample is available in Table 1. The clinical presentation of the disease varied between patients, but all were at an early stage of the disease (Hoehn&Yahr ≤2). Participants were also screened for cognitive deficits using the Montreal Cognitive Assessment questionnaire (MoCA) and none exhibited a physical limitation or pain affecting their ability to perform the tasks. The study was approved by the Centre de Recherche de l’Institut Universitaire de Gériatrie de Montréal ethics board and written consent were obtained for all participants.

**Table 1:** Sample description.

	Older adults	Parkinson’s disease patients
*n*	16	15
Age	69.1 ± 7.5 years old	65.8 ± 8.4 years old
Gender	50%♀ 50%♂	40%♀ 60%♂
Height	161.3 ± 8.4 cm	171.1 ± 8.3 cm
Weight	63.2 ± 10.1 kg	74.1 ± 18.2 kg
BMI	24.3 ± 3.2 kg/m^2^	25.5 ± 6.8 kg/m^2^
H&Y*	–	H&Y = 1:47%
		H&Y = 2:53%
Nb years since diagnostic	–	5.7 ± 4.9
Montreal Cognitive Assessment questionnaire	–	27 ± 3
Levodopa equivalent daily dose	–	508 ± 165 mg/day
Remaining symptoms^a^		
Tremors	–	27% yes; 73% no
Rigidity	–	33% yes; 67% no
Bradykinesia	–	7% yes; 93% no

*All values are reported as mean and SD unless otherwise stated*.

*^a^Frequencies, under medication (*ON*)*.

### Experimental Protocol

**Figure 3:**
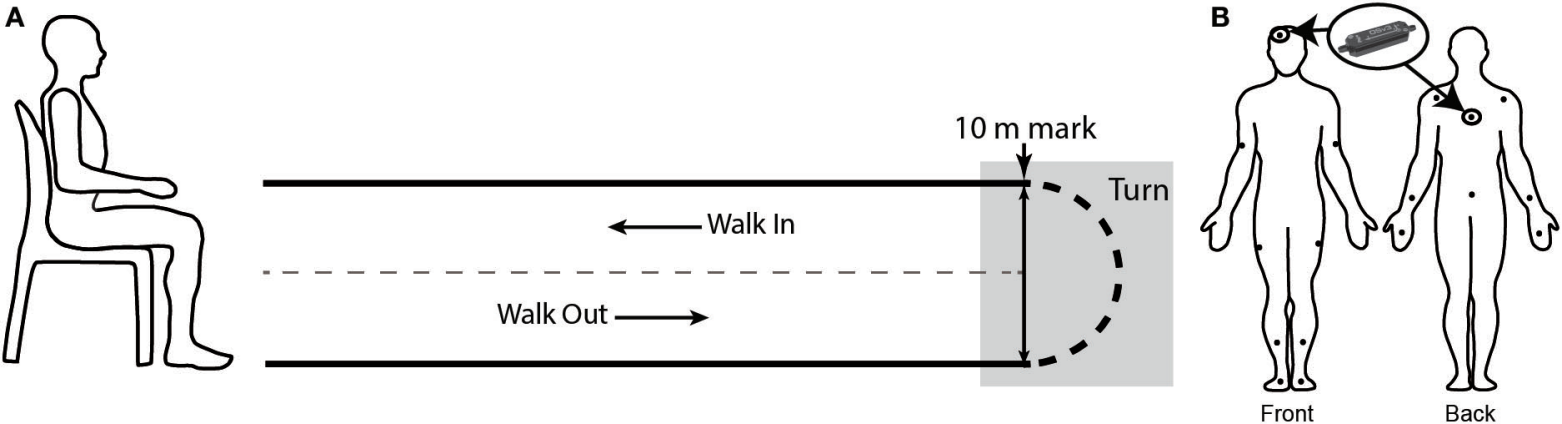
Experimental protocol. **(A)** timed up-and-go schematic. Participant was initially seated on a chair. Upon signal, the participant stood-up, walked up to the 10 m mark, turned, and came back to his initial position, on the chair. The turn phase was manually segmented in post-processing. **(B)** Participants were equipped with a suit comprised of 17 AHRS. Orientation data from the sensors placed on the head and the trunk were further processed for cranio-caudal signature analysis.

Participants performed repeated 10 m timed up-and-go (TUG) trials (Figure 3A) while outfitted with the IGS-180 suit (Synertial Ltd., UK) which contains 17 inertial modules (OS3D, Inertial Labs, USA) enabling full-body kinematics to be captured. Inertial modules positioned on the head and the trunk provided the orientation of both segments during the task (Figure 3B), allowing the computation of the relative orientation of the head to the trunk and the relative angular velocity profile required for cranio-caudal signature assessment. OA participants performed repeated 10 m TUG trials at varying pace (preferred vs fast pace) while patients performed repeated 10 m TUG trials at preferred speed both in their *OFF* and *ON* states. *OFF* state was defined as a minimum delay of 10 h since their last medication (PDoff) while *ON* state corresponded to a minimum delay of 45 min after medication intake, when motor symptoms disappeared or were greatly reduced (PDon), as confirmed by the patients themselves.

Each trial was manually segmented using a set of criteria defined in Lebel et al. (17) to isolate the turn phase. Briefly, the initial misalignment of the head–trunk–pelvis axis was visually identified. The point in time where the previous gait cycle began (i.e., heel strike) was defined as the beginning of the turn. Then, the point in time where the head–trunk–pelvis realignment occurred was established, again through visual inspection. Heel strike of the following gait cycle was defined as the end of the turn. Segmentation was performed by the same evaluator in an attempt to minimize potential bias.

### Signal Processing and Statistical Analysis

Cranio-caudal signature assessment follows the process described in Figure 1. Briefly, the relative orientation of the head to the trunk during the turn phase and its associated relative angular velocity profile are computed from the orientation signals provided by the inertial modules positioned on the head and the trunk of the participant. The head to trunk relative orientation signal is then further processed to identify the maximum angle reach during the turn while the relative angular velocity profile is analyzed through the sigma-lognormal approach, as explained in Section “Turn Cranio-Caudal Kinematic Signature from IS.” For each trial, a set of cranio-caudal metrics characterizing the cranio-caudal pattern is derived (Table 2). All signal processing is performed in Matlab v2015a (MathWorks, USA).

**Table 2:** Turn cranio-caudal signature metrics.

Metric	Description
H2Tmax	Maximal head to trunk angle reached during turn
D_1_, D_2_	Amplitude of the commands for phases 1 and 2 of turn
t_01_, t_02_	Time occurrence of the commands (phases 1 and 2)
$\bar{t_{1}},\;\bar{t_{2}}$	Time delay of the system impulse response (phases 1 and 2)
s_1_, s_2_	Neuromuscular system response time (phases 1 and 2)

For each participant and condition (i.e., healthy, PDon, PDoff), the parameters mean was computed over the repeated trials. The maximum relative angle (H2Tmax) and the amplitude of the NMS command for both phases of the turn (D1, D2) were further analyzed to verify their potential to discriminate between populations (i.e., Healthy vs PDoff) using a Wilcoxon–Mann–Whitney test and receiver operating characteristic (ROC) curves. The sensitivity to change of those same parameters was also evaluated (i.e., PDoff vs PDon), this time using a Wilcoxon signed-rank test. All statistical analysis considered a significance level of 0.05 and were conducted using SPSS (v23.0.0, IBM). Due to the exploratory nature of the study, no correction for multiple comparison was considered.

### Turn Kinematics Traditional Parameters

For comparison purposes, metrics based on raw inertial signals found in the literature, namely the number of steps required to perform the turn, the mean turn velocity, and the max turn velocity, were also computed for each trial. The number of steps is obtained through analysis of the accelerometers signals from the IMUs located on the feet. Briefly, the technique consists in using the norm of the accelerometer signal on which is applied an aggressive high-pass filter to remove most of the signal’s content, leaving only the impacts on the floor. The location of these impacts are then found using a peak detection algorithm. Quality control checks were performed periodically to ensure the accuracy of the results. The mean and max turn velocities correspond to the mean and the max recorded angular velocity of the trunk, in the plane of rotation.

## Results

**Figure 4:**
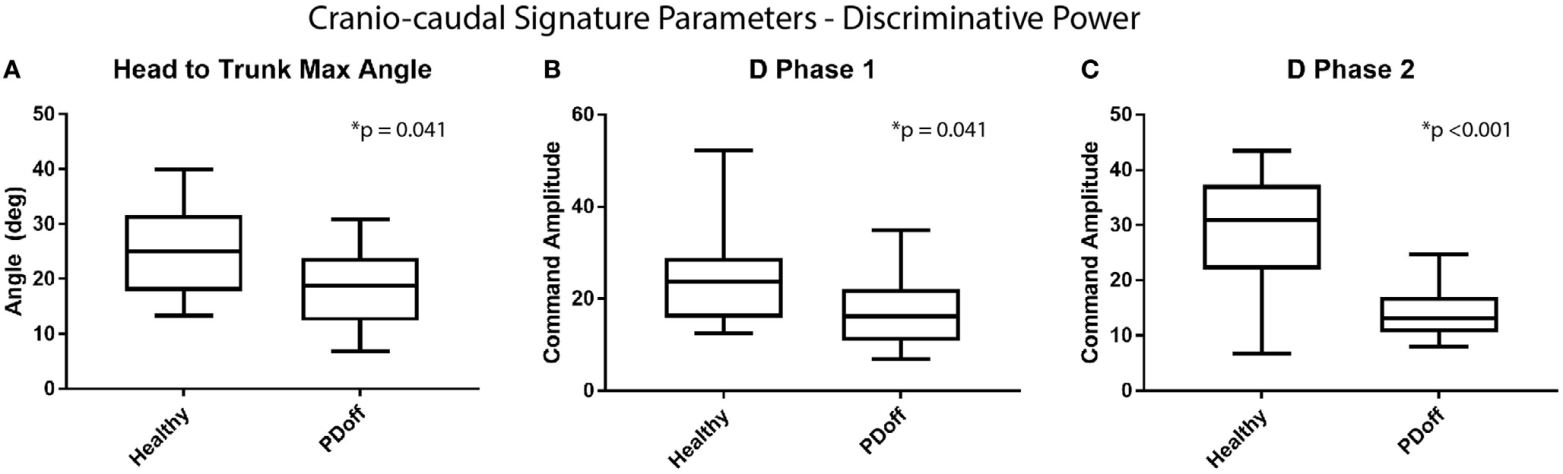
Discriminative power of cranio-caudal signature parameters. Comparison of the distribution in **(A)** head to trunk angle and **(B,C)** amplitudes of the NMS commands for phase 1 and 2 of the turn for healthy elderly vs patients OFF medication.

**Figure 5:**
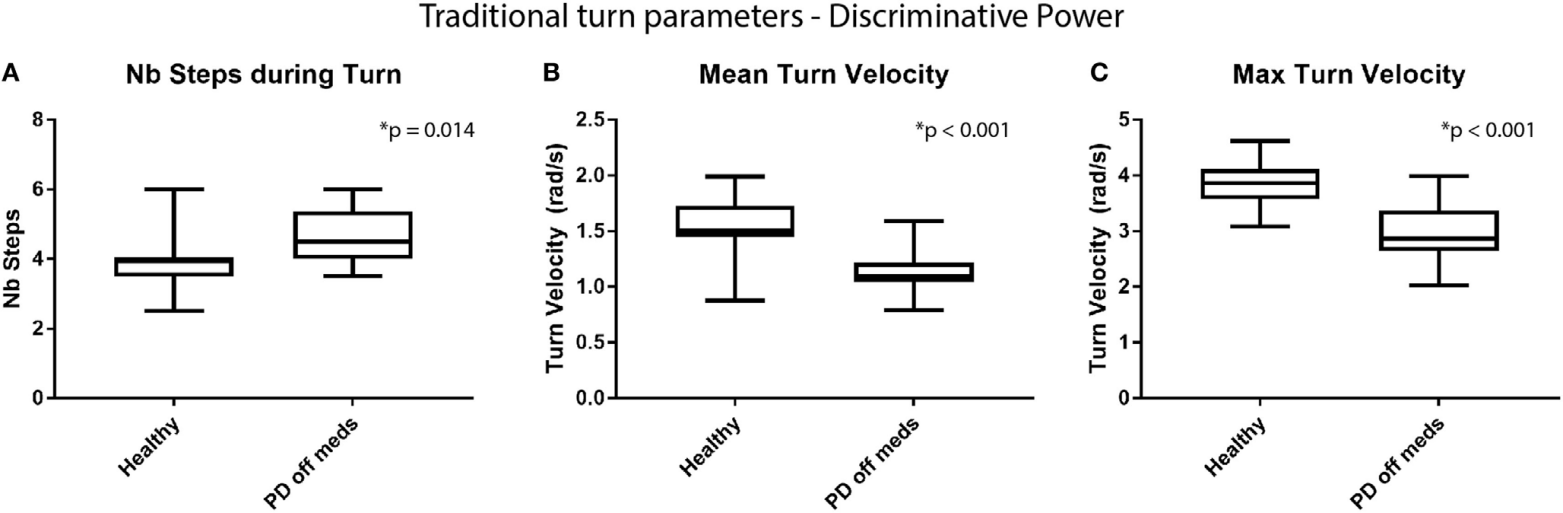
Discriminative power of traditional turn parameters. Comparison of the distribution in **(A)** number of steps required to perform a turn, **(B)** the mean turn velocity, and **(C)** the maximum turn velocity for healthy elderly vs Parkinson’s disease (PD) early stage of PD patients off medication.

The study first aims at verifying the ability of the metrics to discriminate between healthy OA and early PD patients. The distribution of the cranio-caudal parameters value is, therefore, illustrated in Figure 4. Figure 4A shows that the maximum head to trunk angle (H2Tmax) is significantly reduced in patients with early PD compared to OA [healthy (median and inter-quartile range): 25.0° (18.1°; 30.7°), PD: 18.8° (12.5°; 23.7°); *p* = 0.041]. A similar behavior is also observed with both phase 1 and phase 2 NMS commands [D1Healthy: 23.7 (17.1, 28.3), D1PDoff: 16.2 (11.9, 21.5), *p* = 0.041; D2Healthy: 31.0 (22.3, 36.4), D2PDoff: 13.1 (11.4, 15.5), *p* < 0.001], as shown in Figures 4B,C. A ROC curve analysis has also revealed fair to good areas under the curve for the three CCKS metrics [H2Tmax AUC: 0.724 (0.537, 0.911); D1 AUC: 0.724 (0.537, 0.911); D2 AUC: 0.886 (0.748, 1.0)]. As far as the traditional metrics are concerned (Figure 5), the number of steps required to complete the turn has shown a significant increase over the same participants and trials [healthy: 4.0 (3.5, 4.0), PDoff: 4.5 (4.0 5.2); *p* = 0.014] while the turn mean and max velocities have shown a significant decrease [mean turn velocity—healthy: 1.5 rad/s (1.5,1.7), PDoff: 1.1 rad/s (1.1, 1.2), *p* < 0.001; max turn velocity—healthy: 3.9 rad/s (3.6, 4.1), PDoff: 2.9 rad/s (2.8, 3.3), *p* < 0.001].

**Figure 6:**
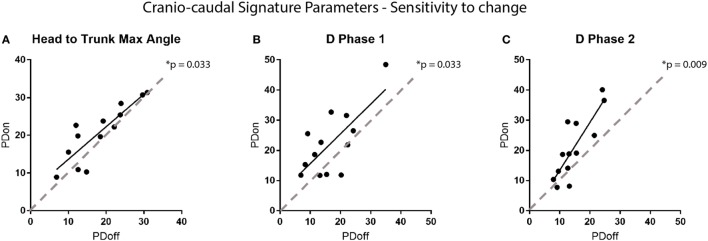
Sensitivity to change of the cranio-caudal signature parameters. Each point on the graphs represents a participant. The graphs show the change in **(A)** head to trunk maximum angle, **(B,C)** amplitudes in NMS commands for phase 1 and 2 of the turn according to the medication state of the participant (i.e., on vs off medication). Dotted lines are the equality line, representing no change in the parameters.

**Figure 7:**
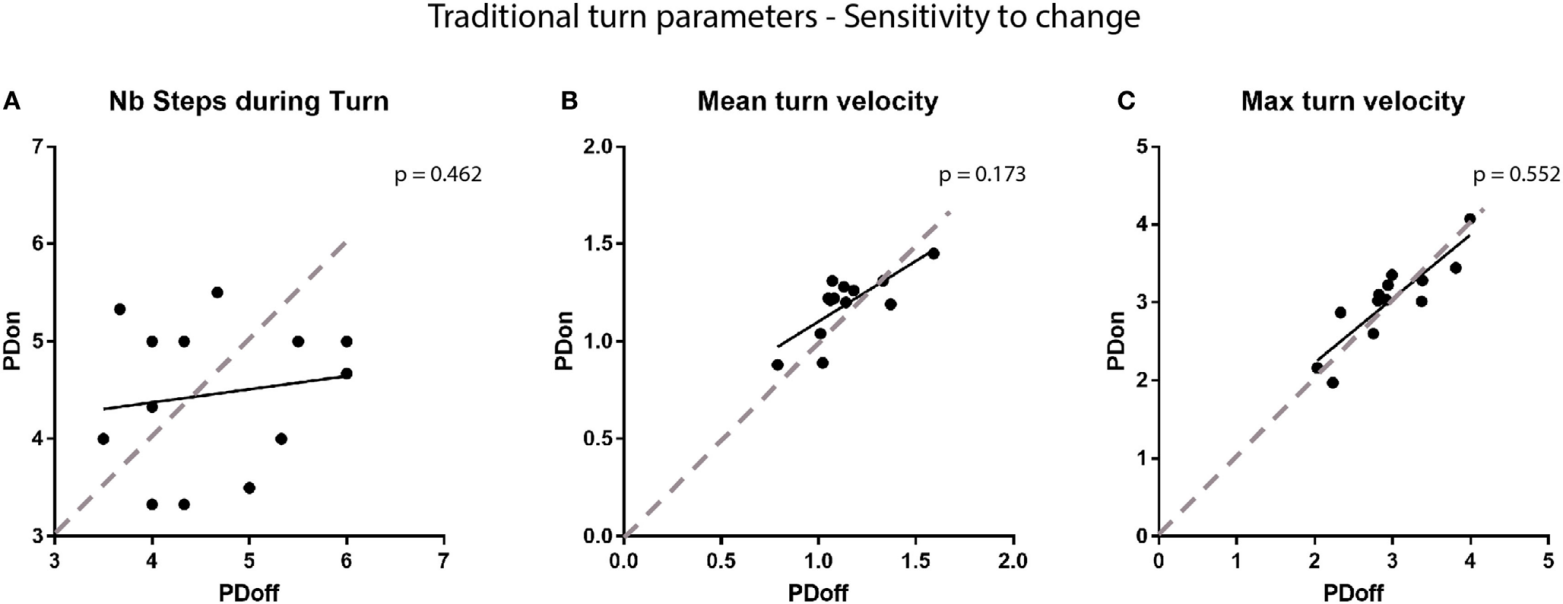
Sensitivity to change of traditional turn parameters. Comparison of **(A)** the number of steps required to complete a turn, **(B)** the mean turn velocity, and **(C)** the maximum turn velocity with the participants’ medication state. Each point on the graphs represents a participant. Dotted lines represent the line of equality, symbolizing no change in the parameters.

Sensitivity to change of the metrics was then verified, comparing results from patients trials performed *ON* vs *OFF* medication. Analysis reveal a statistically significant improvement of the H2Tmax (*p* = 0.033) and the NMS command amplitudes (D1: *p* = 0.033, D2: *p* = 0.009) with medication (see Figure 6). However, traditional metrics did not capture a significant change with medication in either the number of steps required to complete the turn, the mean turn velocity or the max turn velocity (number of steps: *p* = 0.462, mean turn velocity: *p* = 0.173, max turn velocity: *p* = 0.552), as shown in Figure 7.

## Discussion

A previous study performed by our team had shown the ability of the proposed methodology to capture the turn cranio-caudal signature in asymptomatic elderly (17). The current study goes one step further in the validation process, exploring the ability of the methodology to capture the turn cranio-caudal signature in patients with PD. General signature pattern was similar for both populations, but the signature characteristics were different (e.g., amplitude). Indeed, the three metrics derived from the signature (i.e., maximum head to trunk angle and amplitudes of the NMS commands for both phases of the turn) have shown a potential in discriminating between healthy individuals and patients, with all three parameters having significantly reduced values in patients. The metrics also appear to be sensitive to medication change, enabling to discriminate between the patients’ medication states. Indeed, CCKS metrics were, on average, higher when patients were ON dopaminergic therapy. In early states of PD, dopamine depletion mainly occurs at the dorsal striatum level, affecting planning and execution of tasks. It can, therefore, be hypothesized that the captured variation in CCKS metrics (i.e., reduced values in PD compared to healthy elderly and improved values on dopaminergic therapy for patients) can be linked to the efficacy of the dopaminergic pathways. For comparison purposes, the number of steps taken while turning as well as the maximum and mean angular turn velocities were investigated for the same participants and trials. These more traditional metrics have also shown a good ability to discriminate between populations, in accordance with the literature (19). However, they were not able to differentiate between medication states, indicating a lower sensitivity to change. This inability of traditional metrics to differentiate between medication states in early stages of PD was also noted by Curtze et al. (20). In a recent review on turn deficits in PD, Hulbert et al. (14) proposed to categorize turn deficits into axial, defined as an impaired movement of the head–trunk rotational axis, and perpendicular, referring to suboptimal movement in the limbs. The authors suggest that axial deficits may occur earlier, leading to altered control in perpendicular segments. From this perspective, the number of steps required to perform a turn relates to perpendicular deficits while CCKS turn metrics are linked to axial deficits. Hence, the results from this study tend to endorse Hulbert et al.’s (14) hypothesis that axial deficit may come earlier in PD, therefore offering a more sensitive measure of the mobility impairments.

The signal processing technique used in the CCKS approach is partly based on the use of the sigma-lognormal model to provide insights into the NMS. Although well known to analyze movement in translation over a single segment, the CCKS is the first application to consider the model on a complex system (i.e., multiple segments) as well as on advanced orientation data. The interesting results reported in the original method paper as well as in the current manuscript reveal a potential for the model to be used in this new context. Furthermore, the methodology is based on inertial sensors, a technology that has the major advantage of being portable and relatively low-cost. Hence, the proposed cranio-caudal kinematic signature technique has the potential to be used in the clinic to enhance the accessibility to objective turn mobility assessment. Combined with autonomous activity identification and segmentation algorithms (21), the technic even has the potential to be used in-home to assess mobility of patients in their natural environment.

A limit to this study is the relatively small sample size. However, all patients recruited were in early stages of the disease to avoid extreme motor symptoms related to later stages of the disease. In other words, the CCKS was tested in the most stringent conditions since later stages of the disease would have probably exacerbated the motor deficits in the OFF condition, making it easier for our algorithms to detect them. Nevertheless, further validation of the metrics on a larger sample including a wider spectrum of symptoms is required to enable a clinical understanding of the variations in the metrics. Furthermore, the small sample size combined with the exploratory nature of the study has driven the statistical plan of analysis which does not include a correction for multiple comparison. Indeed, this approach was chosen in order to remain open-minded in the identification of the potential of CCKS metrics and avoid to inflate type-II error (22). Future studies to confirm the potential of CCKS metrics will, however, address this issue.

## Conclusion

The present study reveals a potential for CCKS to assess mobility impairments and medication state at initial stages of the disease. The wearable IS used in this study combined with the sensitivity to change of the CCKS metrics opens the possibility for this technic to be used as markers for disease progression or to assess impact of medication on mobility impairments.

## Ethics Statement

Participants gave their informed consent following the procedure approved by the Centre de Recherche de l’Institut Universitaire de Gériatrie de Montreal ethics board.

## Author Contributions

KL developed the algorithm, designed the analysis, and drafted the manuscript. CD conceived the experiment, helped in data interpretation, and reviewed the paper. HN provided significant feedback on the analysis of the study and the manuscript. RP provided feedback on the use of the Sigma-Lognormal model and its interpretation and reviewed the manuscript. PB helped in the conception of the algorithm, the interpretation of the data, and reviewed the analysis and the manuscript.

## Conflict of Interest Statement

The authors declare that the research was conducted in the absence of any commercial or financial relationships that could be construed as a potential conflict of interest.
